# The Influence of Sociodemographic Factors, Lifestyle, and Risk Perception on Dietary Patterns in Pregnant Women Living in Highly Contaminated Areas: Data from the NEHO Birth Cohort

**DOI:** 10.3390/nu14173489

**Published:** 2022-08-25

**Authors:** Silvia Ruggieri, Gaspare Drago, Simona Panunzi, Giulia Rizzo, Elisa Eleonora Tavormina, Sabina Maltese, Fabio Cibella

**Affiliations:** 1 Institute for Biomedical Research and Innovation, National Research Council of Italy, 90146 Palermo, Italy; 2 Institute for System Analysis and Computer Science—BioMatLab, National Research Council of Italy, 00168 Rome, Italy

**Keywords:** dietary patterns, pregnancy, principal component analysis, socioeconomic factors, lifestyle, public health

## Abstract

During pregnancy, maternal nutrition and lifestyle play a critical role in influencing fetal development and newborn health outcomes. The aim of this study is to investigate the factors influencing the adherence to dietary patterns in pregnant women living in highly contaminated areas, and whether women with higher environmental risk perception manifest different nutritional behaviors during pregnancy. Food consumption data on 816 pregnant women from the Neonatal Environment and Health Outcomes (NEHO) residential birth cohort were analyzed. Dietary patterns were computed by principal component analysis. A multinomial logistic regression was also applied to identify sociodemographic, lifestyle, and pregnancy-related determinants of adherence to dietary patterns during pregnancy. Three patterns of food consumption—explaining 24.9% of the total variance—were identified as “prudent”, “high energy”, and “vegetarian” patterns. Results suggest that food choices during pregnancy follow a social gradient and align with other health behaviors during pregnancy: older, better educated, and physically active women with higher risk perception are more likely to follow healthier dietary patterns. Knowledge about what is eaten can contribute to dietary choices. Interventions to improve the prenatal nutrition knowledge of pregnant women are needed, especially concerning younger mothers and those with lower educational levels.

## 1. Introduction

During pregnancy, a multitude of interactions between genetic and environmental factors plays a fundamental role in fetal development [1,2], and, among environmental factors, maternal nutrition plays a critical role in influencing fetal health development as well as the short- and long-term health of the child [3,4,5].

Within this perspective, the developmental origins of health and diseases (DOHaD) theory supports evidence that explains how nutrition in utero and during early childhood influences lifelong health [6]. Moreover, DOHaD indicates that improving the early life environment results in health and resilience over the lifecourse.

Nutritional status during pregnancy is the result of a combination of food and nutrients. In accordance with the Institute of Medicine’s (IOM) guidelines, a healthy diet should be practiced and a variety of foods should be consumed to meet the nutrients and energy demands during pregnancy [7]. In addition to the IOM recommendations, several studies have indicated that maternal dietary habits also appear to be influenced by social determinants and lifestyle factors (i.e., socioeconomic status, educational level, physical activity, and smoking habits) [8,9,10].

In this regard, diet and lifestyle during pregnancy are determinants for child and maternal health [11,12], especially for pregnant women living in proximity to industrially contaminated sites (ICSs). Contaminated sites can be defined as areas hosting or having hosted human activities which have produced environmental contamination of all environmental matrices, including food-chain, and resulting in human health impacts. There are many ICSs in Europe [13] and in Italy; a total of 42 ICSs are officially defined as national priority contaminated sites (NPCSs) for environmental remediation. Living in these areas means being exposed to many environmental risk factors which may produce risks to both adult and children health [14,15,16]. Contaminants such as heavy metals (HMs) and persistent organic pollutants (POPs) may transfer from one environmental matrix to another one and, depending on their chemical-physical properties, are able to infiltrate the human body through different exposure pathways and routes [17]. In a previous study, to better describe pregnant women living in high-risk areas, we evaluated risk perception (RP) during pregnancy, a useful tool for understanding communities living in high-risk areas and preventing dangerous exposures [18]. We found that environmental and health risk perception in pregnant women residing in industrialized areas was higher when compared to risk perception reported from women living in areas with less industrial impact [18].

To our knowledge, no previous studies have evaluated whether a link exists between maternal environmental RP and dietary pattern adherence in pregnant women living in highly industrialized areas, thus the goal of this study was to investigate how sociodemographic factors (i.e., age, education, marital status), and lifestyle/health-related behaviors (e.g., smoking, alcohol use, physical activity, pre-pregnancy body mass index [BMI], weight gain [WG] during pregnancy, use of nutritional supplements) influence dietary pattern adherence in pregnant women living in highly contaminated areas where, in turn, dietary patterns can represent one of the main contaminant exposure pathways [19]. Moreover, we aimed at verifying if mothers’ RP itself is able to influence diet choices during pregnancy. 

To explore these possible relationships, we used data collected from questionnaires filled in by pregnant women participating in the Neonatal Environment and Health Outcomes (NEHO) residential birth cohort [20,21]. Mother-child cohort studies are a suitable tool, and are likely the most advanced instrument we can use for identifying both maternal and child exposure in early life and possible long-term health outcomes in highly contaminated sites [22].

## 2. Materials and Methods

### 2.1. Study Design and Population

The NEHO birth cohort is an ongoing population-based study designed to investigate the effects of sociodemographic, environmental, lifestyle, and pregnancy-related factors on mother-child pairs living in the three NPCSs of Augusta, Crotone, and Milazzo in the Mediterranean area of southern Italy. The NEHO study was activated within the framework of the CISAS (International Centre of Advanced Study in Environment, Ecosystem and Human Health) project, which investigated environmental pollution and its impact on ecosystems and human health in three selected NPCSs: Milazzo-Valle del Mela (hereafter referred to as Milazzo) and Augusta–Priolo (hereafter referred to as Augusta), located in the Region of Sicily, and Crotone, located in Calabria [19,23,24,25,26,27]. The NEHO study enrolled mother-child pairs living in three NPCSs in southern Italy, along with pregnant women living in surrounding areas (local reference areas, LRAs), outside the perimeter of the NPCSs, but presenting similar geographic and sociodemographic characteristics [20,21]. Briefly, pregnant women were invited to participate in the study when admitted to the maternity units of the public hospitals of four cities: the “G. Fogliani” Hospital in Milazzo (for the Milazzo NPCS), the General Hospital of Lentini and the “Umberto I” Hospital in Syracuse (for the Augusta NPCS), and the “San Giovanni di Dio” Hospital in Crotone. Women were enrolled according to certain inclusion criteria: being 18–40 years old at the time of delivery; having no assisted reproduction program; an absence of serious chronic diseases (i.e., diabetes, hypertension, etc.); and an absence of any evident complications during pregnancy, which means in principle not requiring special diets. On the basis of these general inclusion criteria [20], 845 pregnant women were recruited, during the last two months of pregnancy, on a voluntary basis. After inclusion in the study, pregnant women were asked to fill out a questionnaire collecting information on maternal health, lifestyle, and diet habits during the gestational period; 29 mothers were excluded since their questionnaires lacked more than 20% of the responses related to dietary habits during pregnancy, leaving 816 questionnaires for subsequent analysis.

### 2.2. Data Assessment

The questionnaire collected data about lifestyle and health status during pregnancy and was administered from 32th gestational week. The section “Dietary and Alcohol Habits”, concerning information on frequency of food consumption, was based on the questionnaire adopted by *Piccolipiù Birth Cohort*, the largest Italian residential birth cohort [28]. This section has been integrated for including product categories and their origin (i.e., large-scale distribution, local markets, etc.) in order to evaluate food origin and expanded for certain food categories (e.g., fish) for taking into account possible food chain contamination [19]. The questionnaire investigated 41 different types of foods (i.e., pasta, beef, fruit, etc.). In correspondence to each type of food, mothers reported the frequency of its consumption (i.e., never, once a month, once a week, etc.). The frequency of food intake was converted into a quantitative variable using the LARN (National Recommended Energy and Nutrient Intake Levels) as the food standard quantity in grams per portion [29]. The total food consumed was then considered per week. Maternal pre-pregnancy weight and height information were collected in the questionnaire completed at recruitment. Participants’ pre-pregnancy BMI was calculated according to WHO criteria and, following the guidelines, was categorized into four classes: mothers with a BMI less than 18.5 kg/m^2^ were classified as underweight; mothers with a BMI equal to or greater than 18.5 and less than 25 were considered to be normal-weight; mothers with a BMI ranging from 25.0 and less than 30 were considered overweight; mothers with a BMI equal to or higher than 30 were classified as obese [30,31].

In order to include the largest number of mothers in the analysis, values of items related to food consumption (e.g., dairy products and fruits) were imputed. Imputation was performed with a predictive mean matching method. Several foods were considered in a wider category instead of entering the different analyses as individual types of food. For example, the consumed quantities of fresh and dried legumes were summed up and considered as a unique type of food. The same was made for the different types of bakery products and for different types of fruit.

To determine whether pregnant women with higher perceived risks adhered to healthier dietary patterns than those with lower RP, we used risk perception indices computed in a previous study [18]. Risk perception was evaluated by means of four indices: the Hazard Perception Index (HPI), which reflects the mothers’ perceptions regarding the presence or absence of hazards in their residence area; the Exposure Hazard Perception Index (EHPI), representing the perception of being exposed to a danger in the area where the mothers live; the Health Risk Perception Index (HRPI), which reflects the perception of the health risk (that is, the perception that various diseases could occur in their area of residence [allergies, infertility, chronic respiratory diseases, different types of cancer]); and the Risk Perception Index (RPI), representing the perception of both environmental and health risks. The above indices were computed from a total of 22 items related to a subset of questions reported in Section J of the questionnaire. The questions used in the indices’ construction and the detailed procedure for the computation of the RP indices were reported in a previous work [18]. For each participant, the HPI, EHPI, HRPI, and RPI were calculated with a value from 0 to 1, a higher value corresponding to higher RP. Among the 816 women considered in this study, 589 mothers completed Section J of the questionnaire, and for these it was possible to compute the four RP indices.

### 2.3. Statistical Analysis

Descriptive statistics were used to characterize the population characteristics using means and standard deviations to describe quantitative variables; numbers and percentages were used for categorical variables.

Principal Component Analysis (PCA) is one of the most commonly used exploratory methods to derive dietary patterns [32]. The quantitative variables, expressing the consumption of different types of food, entered a PCA for dimensionality reduction with the aim of individuating a set of dietary patterns. Each dietary pattern was identified on the basis of the contributions each item (food) showed on the first three principal components. Mothers were then classified according to the values they present on the three principal components (each one representing a specific dietary pattern) resulting from the PCA by means of the hierarchical clustering on principal components (HCPC) approach. The HCPC combines three multivariate data analyses: principal component methods (such as PCA, correspondence analysis, multiple correspondence factorial analysis for mixed data, multiple factorial analysis), hierarchical clustering, and partitioning clustering (i.e., k-means method).

A multinomial logistic regression model was used to investigate the relationship between population characteristics, such as the socioeconomic level of the enrolled mothers: educational level (three levels: secondary school or lower classification, upper secondary school, degree or higher qualification); age (years); smoking (no/yes); alcohol use (no/yes), and the cluster variable (3 levels). The chosen reference category was Cluster 2, which was composed of mothers with high adherence to the second PCA component associated with a high energy profile (i.e., a dietary profile not recommended during pregnancy). The variables entered into the multivariable multinomial logistic model were those which resulted significant at a *p* level <0.05 in a univariable analysis. The final model was then determined by applying a stepwise elimination procedure in both directions. An analysis of variance (ANOVA) was used to test possible differences in the quantitative variables of interest among the three NPCSs and among the three identified clusters. The ANOVA was also used to test whether differences in RP arose among the three clusters. Tukey post-hoc analyses were used to check for differences between each pair of clusters. Possible associations between categorical variables and NPCSs/clusters were assessed with a Chi-Squared test or with a Fisher’s exact test when appropriate. All of the analyses were considered significant at a *p* level of 0.05. The analyses were conducted in R, version 4.1.3 [33].

## 3. Results

### 3.1. Population Characteristics

Among the 816 participants included in this study, the distribution of mothers in the three sites of interest was: 534 (65.5%) in Augusta, 165 (20.2%) in Crotone, and 117 (14.3%) in Milazzo. The sociodemographic, lifestyle, environmental, and pregnancy-related behaviors of the 816 included mothers are presented in Table 1 and Table 2. In general, the average age was 30.6 ± 5.1 years, with no statistical difference among the three sites; BMI and marital status were also not significantly different. Conversely, the educational level was statistically associated with the site: mothers living in Milazzo more frequently had a higher educational level (36.7%) in comparison with mothers living in Crotone (25.5%) and Augusta (21.9%, *p* < 0.001). Mothers from Crotone showed a lower weight gain (11.2 ± 4.4 kg versus 12.1 ± 4.0 kg and 12.9 ± 3.8 kg for the Augusta and Milazzo sites, respectively, *p* < 0.01). The percentage of mothers consuming alcohol during pregnancy was also statistically lower in the Augusta site (1.3%) with respect to the Crotone (10.1%) and Milazzo (13.7%) sites (*p* < 0.001). The assumption of nutritional supplements was investigated as yes/no response categories (Table 2). Supplements use resulted statistically different among the three sites for folic acid, iron, multivitamin complexes, and natural supplements: mothers living in Augusta showed a greater usage of folic acid and iron while women living in Milazzo referred to prefer multivitamin complexes and natural supplements (*p* < 0.001 for all of the four supplements analyzed). Sports activity was practiced most by mothers from Crotone (12.7%), while it was less practiced by mothers from Augusta (3.7%, *p* < 0.001).

### 3.2. Principal Component Analysis Subsection

**Figure 1:**
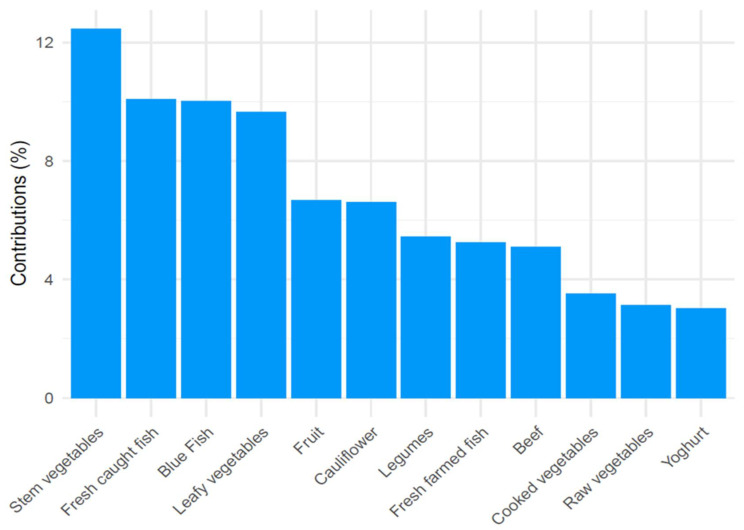
Barplot of contributions to the first principal component from the PCA analysis on food items in the “prudent” dietary pattern. Blue = Positive contribution.

**Figure 2:**
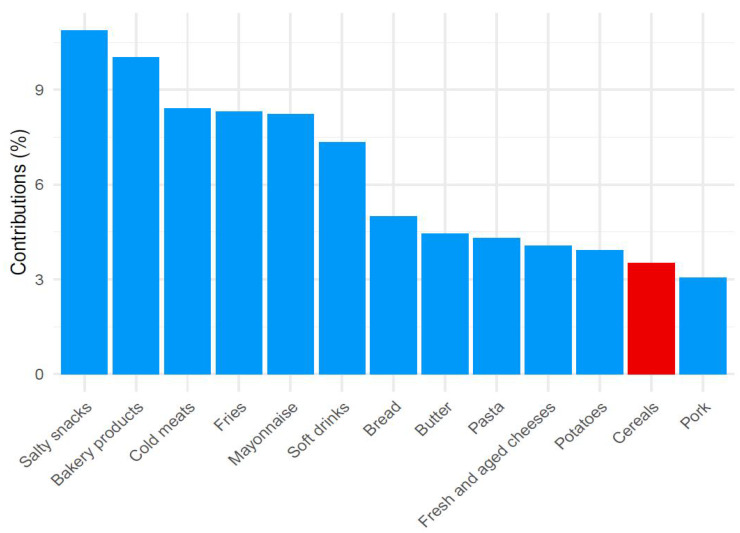
Barplot of contributions to the second principal component from the PCA analysis on food items in the “high energy” dietary pattern. Blue = Positive contribution; Red = Negative contribution.

**Figure 3:**
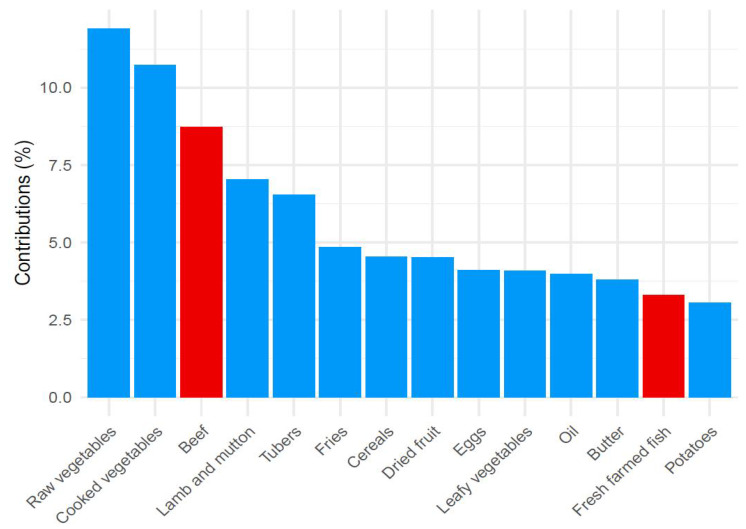
Barplot of contributions to the third principal component from the PCA analysis on food items in the “vegetarian” dietary pattern. Blue = Positive contribution; Red= Negative contribution.

The three components, derived from PCA analysis, represent three different dietary patterns according to the weight that the 38 different types of food entering the analysis showed on the components. The first component was associated with a “prudent” dietary pattern, characterized by a high intake of vegetables, fish, fruit, and beef (Figure 1). The second component was instead interpreted as a “high energy” dietary pattern as salty snacks, bakery products, cold meats, and fries were the foods with higher contributions to the component in conjunction with a negative contribution in correspondence to cereals (Figure 2). The third component was associated with a “vegetarian” dietary pattern since it was characterized by a high intake of vegetables, tubers, and cereals but, on the contrary, with a low intake of meat and fish (Figure 3). These first three components explained 24.9% of the total variance. All of the remaining components explained less than 6% and were therefore discharged. Figure 1, Figure 2 and Figure 3 show the contribution of each item to the first three principal components. Blue bars highlight a positive contribution, whereas red bars show a negative contribution.

### 3.3. Hierarchical Clustering on Principle Components

**Figure 4:**
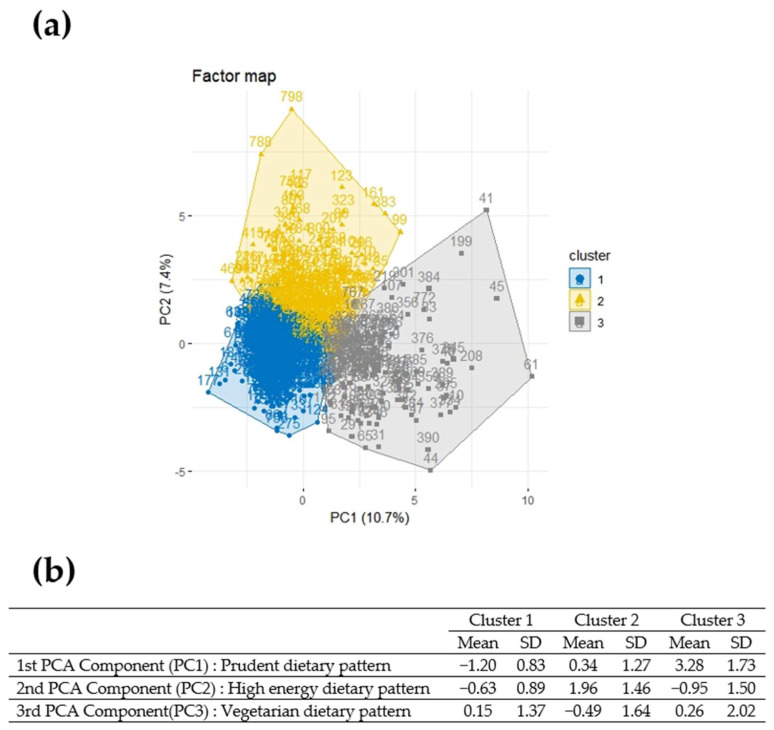
(**a**) Factor map: plot of the individual scores on the first two principal components (PC1, PC2) grouped in the 3 identified clusters. Cluster 1: mainly characterized by high adherence to “Vegetarian” dietary pattern; Cluster 2: mainly characterized by high adherence to “High energy” dietary pattern; Cluster 3: mainly characterized by high adherence to “Prudent” dietary pattern; (**b**) Mean and standard deviations of the three PCA components in the three clusters.

HCPC was carried out on the three components (identifying the three dietary patterns) resulting from the PCA, with the aim of clustering mothers according to their food preferences. Clustering was conducted with k (number of clusters) equal to three. Cluster 1 resulted in 455 (55.8%) mothers and was characterized by mothers with high adherence to the third principal component (“Vegetarian” dietary pattern) but with low values for the first principal component (“Prudent” dietary pattern). Cluster 2 consisted of 217 (26.6%) mothers who showed high adherence to the second principal component (“High energy” dietary pattern). Cluster 3 was composed of 144 (17.6%) mothers with high adherence to the first principal component (“Prudent” dietary pattern). Figure 4 shows the scores of each mother on the first two components and the way they were clustered (panel a) and reports the mean values of the three components in each cluster (panel b).

Table 3 summarizes the maternal characteristics in the three clusters. Mothers in Cluster 3, characterized mostly by women following a “prudent” diet, were older (31.5 ± 4.5 years) than those belonging to Clusters 1 and 2 (*p* < 0.001). There was a statistically significant association between site and cluster (*p* < 0.001), with mothers living in Milazzo mainly belonging to Cluster 3 (38.2%), and most of the mothers from Augusta belonging to Cluster 2 (53.5%). Moreover, Cluster 3 was characterized by a high percentage of mothers with higher qualifications or degrees (36.1%), and the association between cluster and educational level was significant (*p* < 0.001).

Table 4 reports the results related to the association between clusters and the variables characterizing the mothers’ behaviors during pregnancy. Differences in weight gain and smoking were not statistically significant in the three clusters. Conversely, the association between clusters and supplement use (folic acid supplements, iron supplements, multivitamin supplements, and natural supplements) was significant (*p* < 0.001). Folic acid, iron, and multivitamin supplements were mainly assumed by mothers (94.9%) adhering to the “vegetarian” pattern (Cluster 1). The highest percentage of mothers (9.7%) making use of natural supplements was recorded in Cluster 3 (high adherence to the “prudent” pattern), (*p* < 0.001). Finally, mothers who practiced more sport activities were those belonging to Cluster 3 (15.3%), and the association was statistically significant (*p* < 0.001).

Multinomial logistic regressions were carried out to assess any relationship between cluster membership and factors and covariates characterizing the mothers’ lifestyle. The reference level in the multinomial models was Cluster 2 (identifying the “high energy” dietary pattern), as it represented the worst dietary profile in pregnancy.

In a univariate analysis, mothers whose dietary profile was mainly characterized by the “prudent” dietary pattern were more likely to have a higher educational level (High school/Degree or higher qualification) than those who followed a high energy content diet (*p* < 0.001) (Table 3). In addition, as shown in Table 3, mothers who preferred a prudent or vegetarian diet were older than those in the reference level: 29.4 years vs 31.5 (*p* < 0.001) and 30.8 years (*p* = 0.001), in Clusters 1 and 3, respectively, but BMI and weight gain results were not significantly different in the three clusters. As reported in Table 4, alcohol consumption was significantly different in the three clusters, while smoking was not.

In a multivariable model, after a stepwise elimination procedure (in both directions), the final model included three variables: educational level, age, and alcohol consumption. This model (Table 5) confirmed the results of the univariable models, even though smoking did not appear to be a significant predictor any more, possibly due to its association with alcohol consumption (*p* = 0.07 from Fisher’s exact test).

### 3.4. BMI and Weight Gain

**Figure 5:**
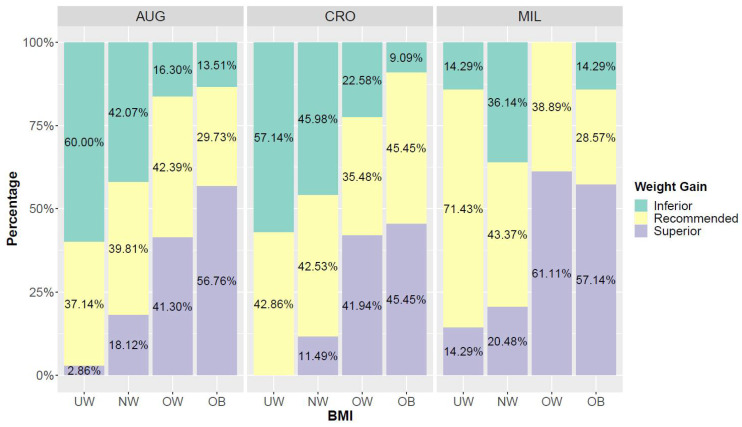
Percentages of mothers who gained Inferior, Recommended or Superior weight according to pre-gravidic BMI classes in the three sites. UW = Underweight, NW = Normal-weight, OW = Overweight, OB = Obese. AUG: Augusta, CRO: Crotone, MIL: Milazzo.

According to WHO criteria and, following the guidelines recommending ranges of weight gain during pregnancy, in correspondence to different pre-pregnancy BMI classes (underweight, normal-weight, overweight, obese), we identified the distribution of mothers’ weight gain for each pre-pregnancy BMI category in the three NPCSs of Augusta, Crotone, and Milazzo [30,31]. Figure 5 shows the distribution of weight gain for each pre-pregnancy BMI category in the three NPCSs of Augusta, Crotone, and Milazzo. Underweight and normal-weight mothers tended to gain less weight than recommended; conversely, mothers in the overweight and obese classes gained more weight during pregnancy. The Cochran-Mantel-Haenszel test results were highly significant (*p* < 0.001) when controlling for the site variable. Partial Fisher’s exacts tests were all significant: *p* < 0.001, *p* = 0.003 and *p* < 0.001 for Augusta, Crotone, and Milazzo, respectively.

**Figure 6:**
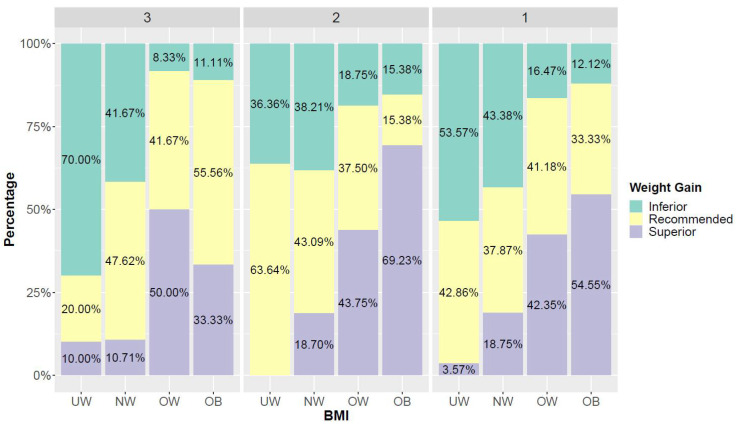
Percentages of mothers who gained Inferior, Recommended or Superior weight according to pre-gravidic BMI classes in the three clusters. UW = Underweight, NW = Normal-weight, OW = Overweight, OB = Obese. Cluster 1: mainly characterized by high adherence to “Vegetarian” dietary pattern; Cluster 2: mainly characterized by high adherence to “High energy” dietary pattern; Cluster 3: mainly characterized by high adherence to “Prudent” dietary pattern; b) Mean and standard deviations of the three PCA components in the three clusters.

Figure 6 shows the distribution of mothers on the basis of the weight gained, for each pre-pregnancy BMI category, in each of the three clusters. The majority of underweight mothers in Clusters 1 and 3 tended to gain less weight than recommended, respectively 70.0% and 53.6%, while in Cluster 2 they tended to gain the recommended weight (63.6%). Normal-weight mothers in Clusters 2 (43.1%) and 3 (47.6%) gained the recommended weight, while in Cluster 1 they tended to gain less than recommended (43.4%). Overweight mothers gained more weight during pregnancy: 50.0% in Cluster 3, 43.8% in Cluster 2 and 42.4% in Cluster 1. In Clusters 1 and 2, obese mothers gained more weight than recommended, respectively 69.2% and 54.6%, while obese mothers in Cluster 3 tended to gain the recommended weight during pregnancy. The Cochran-Mantel-Haenszel test results were highly significant (*p* < 0.001) when controlling for the cluster variable. Partial Fisher’s exacts tests were all significant: *p* < 0.001, *p* < 0.001 and *p* < 0.001 for Clusters 1–3, respectively.

### 3.5. Risk Perception

**Figure 7:**
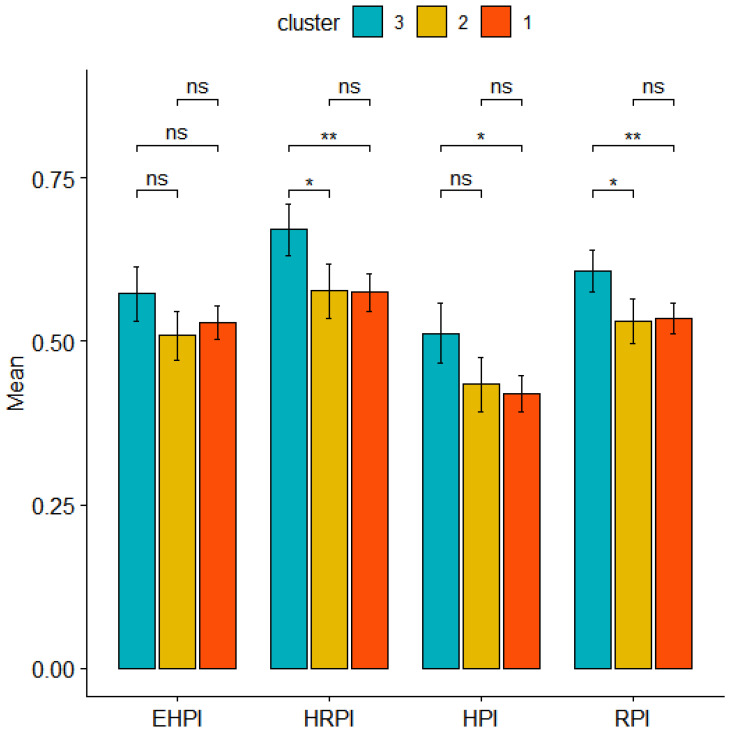
Means and confidence intervals of the four risk perception indices in the three clusters. EHPI: Exposure Hazard Perception Index; HRPI: Health Risk Perception Index; HPI: Hazard Perception Index; RPI: Risk Perception Index. ns: *p* > 0.05; *: *p* < 0.01; **: *p* < 0.001. Cluster 1: mainly characterized by high adherence to “Vegetarian” dietary pattern; Cluster 2: mainly characterized by high adherence to “High energy” dietary pattern; Cluster 3: mainly characterized by high adherence to “Prudent” dietary pattern.

Pregnant women’s environmental risk perceptions were investigated. Average values, confidence intervals, and statistical differences among clusters for the RP indices HPI, EHPI, HRPI, and RPI are shown in Figure 7. Cluster 3 presented the highest average values for all of the four RP indices. For EHPI, no significant difference appeared among the three clusters. HRPI and RPI were statistically significant in the pairwise comparisons between Cluster 3 and the other two clusters. HPI was significantly different between Cluster 3 and Cluster 1.

## 4. Discussion

There are several reasons to investigate dietary intake among pregnant women in highly polluted areas. Analysis of factors that influence food selection during pregnancy is essential for fetal health development [34]. Moreover, assessing dietary patterns of pregnant women living in highly polluted areas, by means of detailed information on consumption of specific foods (i.e., fish, meat, etc.), is crucial for the estimation of possible maternal and fetal exposure to environmental contaminants [19,35,36].

In this study, we identified three distinct dietary patterns based on data collected in a questionnaire completed during the last trimester of pregnancy. Participants with a higher level of education lived in Milazzo, while those with a lower educational level resided in Augusta, as shown in our previous study [20,21]. Although much evidence exists that educational level influences pregnancy behaviors [8,9], when evaluating mothers’ behaviors during pregnancy, the mothers of Augusta were those with the lowest weight gain, lowest alcohol consumption, folic acid and iron supplement use during pregnancy, even though sports activities were practiced more by mothers from Crotone and less by those from Augusta (*p* < 0.001). Following a multinomial logistic regression analysis, sociodemographic, lifestyle, and pregnancy-related determinants of dietary pattern adherence were determined. Older age and higher educational level were significant determinants in adhering to a healthier diet during pregnancy. Moreover, physically active women with higher risk perception were more likely to follow healthier dietary patterns.

Dietary evaluation, with a multitude of foods and drinks consumed every day in varying quantities, is challenging. Due to its complexity, diet is methodologically difficult to capture and no gold-standard method exists [37]. Growing evidence about the limitations of examining single foods or single nutrients led to the concept of dietary patterns [38]. In recent years, nutritional epidemiology, focusing on the contribution of the entire diet, has gradually changed its focus from single nutrients to dietary patterns which have the advantage of taking into account the relationship between different foods and nutrients as a whole. They also provide an alternative and complementary approach to the examination of the relationship between diet and diet-related risks [39,40].

In the literature, dietary patterns have been computed using two different approaches: an a priori approach, analyzing data by using predefined combinations of foods, such as a dietary index, and including dietary scores (e.g., Healthy Eating Index, Recommended Food Score, and Diet Quality Index), also evaluating the conformity of the diet to nutrition guidelines; or an a posteriori method based on exploratory data by cluster and factor analyses [41,42,43].

Among these, a posteriori methods are more commonly used and PCA is a valid exploratory method to derive dietary patterns in which nutritional variables are reduced to a smaller number of variables [31,44]. Moreover, a posteriori dietary patterns have also been calculated as a proxy for several determinants capable of influencing individual food consumption, including social and cultural factors [45,46]. Following an a posteriori method, three patterns of consumption were identified: the “prudent” pattern, the “high energy” pattern and the “vegetarian” dietary pattern. Previous studies suggest that dietary profiles are different in different population and may vary with age, socioeconomic status, ethnicity, lifestyle, cultural traditions, and food availability [8,9,10,47,48]. In order to better describe dietary patterns, we performed an HCPC analysis to identify groups of mothers with similar food preferences, on the basis of the results of PCA analysis. The HCPC analysis identified three different clusters in which enrolled women with similar dietary habits were distributed. The first cluster, as well as the largest, was characterized by mothers with high adherence to the “vegetarian” dietary pattern. Cluster 2 was constituted by mothers with high adherence to the “high energy” dietary pattern. Cluster 3 was represented by mothers with high adherence to the “prudent” dietary pattern.

Our analysis shows that mothers in Cluster 3, characterized mostly by women following a “prudent” diet, were older (*p* < 0.001), with higher qualifications or degrees (*p* < 0.001), mainly living in Milazzo (*p* < 0.001), and practicing more sports activities (*p* < 0.001). On the contrary, mothers belonging to Cluster 2 were younger than the other mothers (*p* < 0.001).

Multinomial logistic regression analysis was applied to identify sociodemographic, lifestyle, and pregnancy-related determinants of dietary pattern adherence during pregnancy. Our results show a social gradient by means of which older and more educated women were more likely to follow healthier dietary patterns. In particular, the profiles of the mothers included in Cluster 3, showing high adherence to the “prudent profile”, were characterized by older women with higher educational qualifications. Moreover, the adherence to the “vegetarian” dietary pattern was a protective factor for the risk of smoking and alcohol consumption. By contrast, the adherence of women to the “high energy” pattern was associated with decreasing age and higher percentage of low-educated women. These results are in line with previous studies [49]. In 2017, Doyle et al. published a systematic review highlighting that food choices during pregnancy follow a social gradient and aligned with other health behaviors during pregnancy (with older, better educated, and physically active women being more likely to follow healthier dietary patterns) [37].

The adherence to the “prudent” pattern is the healthiest choice during pregnancy and foods such as fish and meat play a key-role in the human diet providing proteins, vitamins, and other important nutrients with potential health benefits on maternal health. On the other side, in heavily polluted areas, this may represent the principal pathway of exposure to potentially dangerous compounds, such as heavy metals (HM) and persistent organic pollutants (POPs) [19,50]. In fact, previous studies showed that older women with higher educational level, such as mothers adhering to the “prudent” pattern, had infants with higher POPs serum concentrations than younger women, with lower education [51]. The increase of these values in newborns from mothers with high education levels suggests that different maternal diet profiles may be at the origin of differences in pollutant body burden [51]. Similarly, sociodemographic factors and food choices are associated with metals and metalloids concentration analyzed in pregnant women as shown in a recent study [52].

Among sociodemographic and lifestyle-related factors, educational levels, smoking status, physical activity, pre-pregnancy BMI, and weight gain were the most frequently evaluated [53]. Although there is much evidence that pre-pregnancy BMI is negatively associated with weight gain and diet quality during pregnancy, we did not observe a statistically significant impact on food choices [54,55,56]. Information on dietary supplement use during pregnancy is largely lacking [57]. In this work, we evaluated supplement use by questionnaire answers. In the NEHO birth cohort, women who used supplements during pregnancy significantly differed with respect to their dietary choices from those who did not. Women adhering to the “high energy” profile were those who consumed the least supplements during pregnancy, while the “prudent” dietary pattern was characterized by a high consumption of natural supplements, and the “vegetarian” pattern by a high consumption of folic acid, iron, and multivitamin supplements.

A low adherence to dietary guidelines or nutritional recommendations during pregnancy can have a negative impact on pregnancy and birth outcomes, as well as the future health of offspring [58,59]. A good BMI before conception and an optimal weight gain during pregnancy enhance birth outcomes and reduce pregnancy complications, as suggested by IOM guidelines [33]. In our work, the association between pre-pregnancy BMI and dietary patterns in pregnancy was not statistically significant. In addition, weight gain during pregnancy was not a significant determinant of dietary patterns (*p* > 0.05). To evaluate the behavior of participants, we analyzed weight gain in pregnancy in the three NPCSs taking into account pre-pregnancy BMI: we found that women being under- or normal-weight tended to gain less weight than recommended, in contrast with mothers in the overweight and obese classes who gained more weight during pregnancy (Figure 5). Moreover, when we investigated whether there were weight gain differences between women adhering to the different dietary patterns, we found that overweight and obese mothers gained more weight during pregnancy (Figure 6). In particular, obese mothers adhering to the “high energy” profile gained more weight than recommended. These results are in line with other studies showing that women who were overweight or obese prior to pregnancy were significantly more likely to exceed weight guidelines [33,60,61]. However, women who are overweight or underweight before pregnancy have a higher health risk than those with normal weight [7,62,63]. Moreover, a recommended pre-pregnancy BMI and a correct weight gain are associated with a low risk of fetal and maternal complications in women of normal weight [33,64,65,66,67], especially in highly polluted areas where many studies showed an association between BMI and WG during pregnancy and neonatal exposure to POPs and HM [50,51,68].

To better evaluate the social determinants which influence dietary pattern choices, we also investigated the environmental RP of the pregnant women living in NPCSs to verify whether a higher RP influences food choices during pregnancy. Usually, studies on mothers’ RP refer to risks directly related to motherhood and childbirth, and in most of these works, “perception” is used as a synonym for awareness, recognition, discernment, and understanding. Instead, in a previous study on the mothers of the NEHO birth cohort, we investigated RP related to environmental and health issues, examining the influences of various factors such as measured exposure to hazards, educational level, and perception of personal conditions during pregnancy [18]. In our sample, among the 589 women who answered the questions about RP, we found that the pregnant women adhering to the “prudent” profile presented the highest average values for all of the four RP indices. This result, in line with the previous finding that higher educational qualifications may increase people’s perceptions of environmental risks, suggests that women with higher environmental RP pay more attention to their nutrition during pregnancy. Pregnant women belonging to the “prudent” profile were older and had a higher educational level in comparison to other participants. Consequently, they may be more conscious that they are living a unique moment, during which it is necessary to pay great attention to their own health conditions, engaging in healthy lifestyle behaviors [69,70]. In line with these considerations, having a higher educational level may produce a more conscious approach to one’s diet and to the risk related to the possible presence of environmental pollutants in food. Consequently, this can contribute to dietary choices. Thus, RP during pregnancy is confirmed as being a process influenced by multiple personal, psychological, and societal factors [71]. To our knowledge, no results of specific studies have been published to date on the environmental RP associated with dietary patterns in mother-child cohorts residing in sites with well-known environmental contamination.

### Strengths and Limitations

The strength of the present study is related to its particular geographic and socioeconomic context. In fact, we evaluated healthy pregnant women (without any significant chronic disease), never assisted by reproductive procedures, living in highly contaminated areas. The limitations of the study also need to be mentioned: the recruitment, performed on an exclusively voluntary basis, could have been biased by the similar sociocultural level of the participating women, intercepting different interests toward the health-related aspects of daily living in highly contaminated areas, the cross-sectional nature of the present work, and the lack of validation of the questionnaire collecting information on frequency of food consumption created ad hoc for the study.

## 5. Conclusions

Pregnancy is often considered as a very useful period for improving lifestyle behaviors due to the increased motivation for a healthy pregnancy [72]. Inadequate nutrition during pregnancy remains a public health concern, particularly in high-risk populations living in areas with high environmental pressure. A healthy and varied diet during pregnancy is fundamental, and the importance of maintaining it should not be underestimated. It is highly recommended to understand what women residing in heavily polluted areas eat in order to direct pregnant women to a healthy and balanced diet. Large-scale designed studies during preconception and throughout pregnancy are needed to evaluate the consequences of adherence to different dietary profiles on pregnancy outcomes and related health-life consequences. Moreover, to assess the real maternal exposure pathways to contaminants, further investigations are recommended to clarify this issue. In this context, encouraging pregnant women to meet the recommended IOM guidelines for WG may reduce the accumulation of contaminants in newborns and planning communication activities are particularly important especially for disadvantaged mothers living in areas where a recognized environmental hazard is present, to ensure healthy nutritional choices [73]. Finally, interventions aimed at improving the prenatal nutrition knowledge of pregnant women should primarily target those who are younger and have a lower educational level. Knowledge about what one eats can contribute to dietary choices.

## Figures and Tables

**Table 1:** Mothers’ characteristics by residence site.

Maternal Characteristics	AUGUSTA-PRIOLO (*n* = 534)	CROTONE (*n* = 165)	MILAZZO (*n* = 117)	*p* Value	COHORT (*n* = 816)
Age, years (mean ± SD)	30.4 (±5.1)	30.5 (±5.4)	31.5 (±4.5)	0.12 *	30.6 (±5.1)
	(*n* = 533)	(*n* = 164)	(*n* = 116)		(*n* = 813)
Pre-gravidic BMI, kg/m^2^ (mean ± SD)	23.6 (±4.9)	23.7 (±5.1)	22.9 (±3.7)	0.30 *	23.5 (±4.8)
Educational level				<0.001 **	
Second school or lower classification	153 (28.7%)	40 (24.2%)	12 (10.3%)		205 (25.1%)
High school	264 (49.4%)	83 (50.3%)	62 (53.0%)		409 (50.1%)
Degree or higher qualification	117 (21.9%)	42 (25.5%)	43 (36.7%)		202 (24.8%)
	(*n* = 532)	(*n* = 165)	(*n* = 117)		(*n* = 814)
Marital status				0.11 **	
Unmarried	208 (39.1%)	50 (30.3%)	41 (35.0%)		299 (36.7%)
Married	324 (60.9%)	115 (69.7%)	76 (65.0%)		515 (63.3%)

* *p* values from ANOVAs; ** *p* values from Chi-Squared tests. Significant *p* values are indicated in bold.

**Table 2:** Variables characterizing mothers’ behaviors during pregnancy by residence site.

Pregnancy Behaviors	AUGUSTA-PRIOLO (*n* = 534)	CROTONE (*n* = 165)	MILAZZO (*n* = 117)	*p* Value	COHORT (*n* = 816)
	(*n* = 473)	(*n* = 137)	(*n* = 116)		(*n* = 726)
Weight gain, kg (mean ± SD)	12.1 (±4.0)	11.2 (±4.4)	12.9 (±3.8)	<0.01 *	12.8 (±)
	(*n* = 532)	(*n* = 164)	(*n* = 117)		(*n* = 813)
Smoking				0.60 **	
No	471 (88.5%)	144 (87.8%)	107 (91.5%)		722 (88.8%)
Yes	61 (11.5%)	20 (12.2%)	10 (8.5%)		91 (11.2%)
	(*n* = 533)	(*n* = 158)	(*n* = 117)		(*n* = 808)
Alcohol consumption				<0.001 **	
No	526 (98.7%)	142 (89.9%)	101 (86.3%)		769 (95.2%)
Yes	7 (1.3%)	16 (10.1%)	16 (13.7%)		39 (4.8%)
	(*n* = 534)	(*n* = 165)	(*n* = 117)		(*n* = 816)
Folic acid supplements				<0.001 **	
No	8 (1.5%)	24 (14.5%)	49 (41.9%)		81 (9.9%)
Yes	526 (98.5%)	141 (85.5%)	68 (58.1%)		735 (90.1%)
	(*n* = 534)	(*n* = 165)	(*n* = 117)		(*n* = 816)
Iron supplements				<0.001 **	
No	205 (38.4%)	102 (61.8%)	72 (61.5%)		379 (46.4%)
Yes	329 (61.6%)	63 (38.2%)	45 (38.5%)		437 (53.6%)
	(*n* = 534)	(*n* = 165)	(*n* = 117)		(*n* = 816)
Multivitamin supplements				<0.001 **	
No	211 (39.5%)	149 (90.3%)	42 (35.9%)		402 (49.3%)
Yes	323 (60.5%)	16 (9.7%)	75 (64.1%)		414 (50.7%)
	(*n* = 534)	(*n* = 165)	(*n* = 117)		(*n* = 816)
Natural supplements				<0.001 **	
No	532 (99.6%)	162 (98.2%)	74 (63.2%)		768 (94.1%)
Yes	2 (0.4%)	3 (1.8%)	43 (36.8%)		48 (5.9%)
	(*n* = 534)	(*n* = 165)	(*n* = 117)		(*n* = 816)
Sport activity				<0.001 **	
No	514 (96.3%)	144 (87.3%)	103 (88.0%)		761 (93.3%)
Yes	20 (3.7%)	21 (12.7%)	14 (12.0%)		55 (6.7%)

* *p* values from ANOVAs; ** *p* values from Chi-Squared tests. Significant *p* values are indicated in bold.

**Table 3:** Mothers’ characteristics by cluster.

Maternal Characteristics	CLUSTER 3/ PRUDENT (*n* = 144)	CLUSTER 2/ HIGH ENERGY (*n* = 217)	CLUSTER 1/ VEGETARIAN (*n* = 455)	*p* Value
Age, years (mean ± SD)	31.5 (±4.5)	29.4 (±5.3)	30.8 (±5.1)	<0.001 *
	(*n* = 144)	(*n* = 215)	(*n* = 454)	
Pre-gravidic BMI, kg/m^2^ (mean ± SD)	23.1 (±4.0)	23.3 (±4.6)	23.7 (±5.0)	0.40 *
SIN				<0.001 **
Augusta-Priolo	40 (27.8%)	116 (53.5%)	378 (83.1%)	
Crotone	49 (34.0%)	58 (26.7%)	58 (12.7%)	
Milazzo	55 (38.2%)	43 (19.8%)	19 (4.2%)	
Educational level				<0.001 **
Second school or lower qualification	15 (10.4%)	66 (30.4%)	124 (27.2%)	
High school	77 (53.5%)	110 (50.7%)	222 (48.8%)	
Degree or higher qualification	52 (36.1%)	41 (18.9%)	109 (24.0%)	
	(*n* = 143)	(*n* = 217)	(*n* = 454)	
Marital status				0.08 **
Unmarried	42 (29.4%)	89 (41.0%)	168 (37.0%)	
Married	101 (70.6%)	128 (59.0%)	286 (63.0%)	

* *p* values from ANOVAs; ** *p* values from Chi-Squared tests. Significant *p* values are indicated in bold.

**Table 4:** Variables characterizing mothers’ behaviors during pregnancy by cluster.

Pregnancy Behaviors	CLUSTER 3/ PRUDENT (*n* = 144)	CLUSTER 2/ HIGH ENERGY (*n* = 217)	CLUSTER 1/ VEGETARIAN (*n* = 455)	*p* Value
	(*n* = 127)	(*n* = 180)	(*n* = 419)	
Weight gain, kg (mean ± SD)	11.8 (±3.8)	12.4 (±4.3)	12.0 (±4.0)	0.40 *
Smoking	(*n* = 143)	(*n* = 217)	(*n* = 453)	0.09 **
No	13 (90.9%)	184 (84.8%)	408 (90.1%)	
Yes	13 (9.1%)	33 (15.2%)	45 (9.9%)	
	(*n* = 141)	(*n* = 213)	(*n* = 454)	
Alcohol consumption				<0.001 **
No	128 (90.8%)	198 (93.0%)	443 (97.6%)	
Yes	13 (9.2%)	15 (7.0%)	11 (2.4%)	
Folic acid supplements				<0.001 **
No	31 (21.5%)	27 (12.4%)	23 (5.1%)	
Yes	113 (78.5%)	190 (87.6%)	432 (94.9%)	
Iron supplements				<0.001 **
No	89 (61.8%)	107 (49.3%)	183 (40.2%)	
Yes	55 (38.2%)	110 (50.7%)	272 (59.8%)	
Multivitamin supplements				<0.001 **
No	85 (59.0%)	141 (65.0%)	176 (38.7%)	
Yes	59(41.0%)	76 (35.0%)	279 (61.3%)	
Natural supplements				<0.001 **
No	130 (90.3%)	197 (90.8%)	441 (96.9%)	
Yes	14 (9.7%)	20 (9.2%)	14 (3.1%)	
Sport activity				<0.001 **
No	122 (84.7%)	204 (94.0%)	435 (95.6%)	
Yes	22 (15.3%)	13 (6.0%)	20 (4.4%)	

* *p* values from ANOVAs; ** *p* values from Chi-Squared tests. Significant *p* values are indicated in bold.

**Table 5:** Coefficients and relative *p* values of the predictors retained in the final multinomial model for cluster membership assessment. Reference: Cluster 2 “High energy”.

Variables	CLUSTER 3/PRUDENT		CLUSTER 1/VEGETARIAN	
	Coefficients	*p* Value	Coefficients	*p* Value
Educational level				
High school	1.05	**<0.01**	0.02	0.93
Degree or higher qualification	1.45	**<0.001**	0.19	0.44
Age, years	0.06	**<0.01**	0.05	**<0.01**
Alcohol consumption (yes)	0.13	0.74	−1.20	**<0.01**

Significant *p* values are indicated in bold.

## Data Availability

No applicable.
